# Codeswitching: A Bilingual Toolkit for Opportunistic Speech Planning

**DOI:** 10.3389/fpsyg.2020.01699

**Published:** 2020-07-17

**Authors:** Anne L. Beatty-Martínez, Christian A. Navarro-Torres, Paola E. Dussias

**Affiliations:** ^1^Department of Psychology, McGill University, Montreal, QC, Canada; ^2^Department of Language Science, University of California, Irvine, Irvine, CA, United States; ^3^Center for Language Science, The Pennsylvania State University, University Park, PA, United States; ^4^Department of Spanish, Italian and Portuguese, The Pennsylvania State University, University Park, PA, United States

**Keywords:** codeswitching, language production, speech planning, opportunistic planning, language control

## Abstract

The ability to engage in fluent codeswitching is a hallmark of the flexibility and creativity of bilingual language use. Recent discoveries have changed the way we think about codeswitching and its implications for language processing and language control. One is that codeswitching is not haphazard, but subject to unique linguistic and cognitive constraints. Another is that not all bilinguals codeswitch, but those who do, exhibit usage patterns conforming to community-based norms. However, less is known about the cognitive processes that regulate and promote the likelihood of codeswitched speech. We review recent empirical studies and provide corpus evidence that highlight how codeswitching serves as an opportunistic strategy for optimizing performance in cooperative communication. From this perspective, codeswitching is part and parcel of a toolkit available to bilingual codeswitching speakers to assist in language production by allowing both languages to remain active and accessible, and therefore providing an alternative means to convey meaning, with implications for bilingual speech planning and language control more generally.

## Introduction

Traditionally, the study of codeswitching production and bilingual speech more generally has been carried out within separate disciplines, where cognitive psychologists and psycholinguists have primarily centered on exogenously-cued language switching,^1^ and sociolinguists have focused on the analysis of codeswitching patterns within discourse of members of a given speech community. Formal disciplinary differences aside, one recurrent cross-disciplinary finding is that even when highly proficient bilinguals retain full control over the choice of how to use the two languages, switching is cognitively more demanding or costly than staying in one language (e.g., Gollan and Ferreira, 2009; Fricke et al., 2016; Gollan and Goldrick, 2016; cf. Johns and Steuck, 2018). This finding appears counterintuitive given the ubiquity of codeswitching in many bilingual communities, and thus begs the question of why bilinguals codeswitch in the first place. Here we put forth the proposal, based on quantitative analyses of spontaneous codeswitched speech, that codeswitching serves as a toolkit, or an opportunistic strategy for optimizing task performance in cooperative communication. While previous research has focused largely on the costs that codeswitching brings to language processing (Guzzardo Tamargo et al., 2016; Adamou and Shen, 2017; Beatty-Martínez and Dussias, 2017; Byers-Heinlein et al., 2017; for reviews see Van Hell et al., 2015, 2018), we consider the possible advantages that codeswitching may offer to language producers during bilingual language interactions. Critical to this endeavor is the view that codeswitching offers a unique flexibility that is driven by an interplay of bottom-up and top-down processes, but through which resources from both languages are ultimately recruited to convey speakers’ communicative intentions.

We refer to codeswitching patterns as the tendency to switch at particular syntactic or prosodic boundaries, or as proposed by Torres Cacoullos and Travis (2018) “…of the places where bilinguals can switch, where they prefer to do so” (p. 175; see also Poplack, 1993). It is important to note that bilingual speakers use their languages in different ways, and not all contexts of language use incur the same cognitive demands in speech production (Green and Abutalebi, 2013; Luk and Bialystok, 2013; Green and Wei, 2014). Differences in codeswitching experience can affect not only language abilities (Beatty-Martínez and Dussias, 2017; Valdés Kroff et al., 2018), but have also been proposed to mediate the relation between language and cognitive processes (Beatty-Martínez et al., 2019). Furthermore, while not all bilinguals regularly codeswitch, those who do exhibit usage patterns conforming to community-based norms (Beatty-Martínez et al., 2018; Torres Cacoullos and Travis, 2018; Ramírez Urbaneja, 2019).

Although codeswitching serves a variety of discourse functions, intentions to codeswitch are likely subject to pragmatic, and interactional constraints. Poplack (1987) compared codeswitching behaviors of Spanish-English Puerto Ricans living in New York City to those of French-English bilinguals in Ottawa-Hull, Canada, and observed differences in how the communities engaged in codeswitching. While Puerto Ricans adopted an open discourse mode, opportunistically threading together words and phrases from each language in order to convey the intended meaning, Ottawa-Hull bilinguals maximized the salience of switch points to fulfill rhetorical functions such as contrast and emphasis (see also Myslín and Levy, 2015, for a similar observation with Czech-English bilinguals). Importantly, these findings suggest that bilinguals may plan speech differently as a function of their communicative goals (Gardner-Chloros et al., 2013).

Codeswitching patterns are also constrained by bilingual ability. Whereas highly proficient bilinguals typically favor complex intra-sentential codeswitches and exhibit greater consistency of codeswitching occurrences, less proficient bilinguals tend to limit switching to freely movable constituents (e.g., tag items such as “I mean” or “you know”; Poplack, 1980), and show less voluntary control of their switching behavior (Lipski, 2014). This observation is particularly relevant for bilingual speech planning because it shows that “fluent bilinguals codeswitch because they can, and not because they cannot speak any other way” (Lipski, 2014, p. 24). It follows that a better understanding of the processes that mediate codeswitching requires the consideration of bilinguals’ habits of language use as well as the interactional demands of their language environment.

This paper is not intended as a comprehensive review of the literature on codeswitching. Instead, we attempt to take stock of recent empirical findings from spontaneous language use that highlight how codeswitching enables bilinguals to handle cognitively demanding aspects of speech planning. We first consider the influence of bottom-up processes (i.e., structural priming) in codeswitching behavior, and argue that, while codeswitching may be sensitive to priming, bottom-up processes are ultimately modulated by top-down influences so as to convey speakers’ communicative intentions (Green, 2018). As a first approximation, we provide corpus evidence of our own, focusing on complex noun phrases (NPs) in Spanish-English bilinguals who have extensive codeswitching experience, to exemplify how speaker intentions guide production choices in codeswitched speech. While it is beyond the scope of this article to fully evaluate our proposal, we hope to demonstrate the potential of this approach to highlight the value of naturalistic data and improve our understanding of how proficient bilinguals manage to use their two languages opportunistically in production.

## The Contributions of Bottom-Up Factors in Codeswitching

Speakers’ production choices are not independent of their past experiences, as evidenced by the tendency (commonly referred to as structural persistence or priming) to reuse structures that they have recently produced or comprehended themselves (MacDonald, 2013; Dell and Chang, 2014; Torres Cacoullos and Travis, 2018). Priming effects are widespread in spontaneous speech and have been observed both within individual languages (within-language priming) and cross-linguistically, where producing/hearing a structure in one language increases the probability of producing a related structure in the other language (see Pickering and Ferreira, 2008; Gries and Kootstra, 2017, for reviews). Priming has been proposed as an important mechanism for speech planning, serving a facilitative function in processes related to selection and retrieval (MacDonald, 2013). In the case of bilinguals, priming may provide a unique lens with respect to the strength of associations between cross-linguistic representations and the levels of processing at which cross-language activation can occur.

Priming effects are generally stronger when the prime and target are similar, which has led to the hypothesis that words with overlapping form and meaning across languages (e.g., cognates) may precipitate codeswitching (Clyne, 2003; Broersma and de Bot, 2006; Broersma, 2009; de Bot et al., 2009). The logic is that cognate words can enhance the likelihood of a codeswitch by triggering a relatively high degree of cross-language activation, and in so doing, allowing the language system to switch from output in one language to output in another language. Indeed, cross-language priming effects are generally stronger when there is lexical overlap and shared word order across languages (Kootstra et al., 2010), which is congenial to the idea that linguistic representations vary in their degree of activation in bilingual speech production (Green, 2018). In an analysis of the Bangor Miami Corpus (Deuchar et al., 2014), Fricke and Kootstra (2016) found that priming influenced not only the tendency to codeswitch, but the type of codeswitch as well. Importantly, they observed that other-language words, irrespective of whether they share the same word form, influenced the likelihood of codeswitching.

The Fricke and Kootstra (2016) results illustrate how bottom-up processes influence codeswitching behavior. That said, the scope of these effects in explaining codeswitching behavior is likely limited for a variety of reasons. It should be noted that cross-language priming is weaker in strength and shorter-lived than within-language priming (Schoonbaert et al., 2007; Travis et al., 2017). In a study of coreferential subject priming, Torres Cacoullos and Travis (2018) reported that within-language priming was nearly four times stronger than cross-language priming. This result is also consistent with the observation of Myslín and Levy (2015) that words are generally more likely to reoccur in the language of most recent mention. Second, it has been established that speakers’ tendency to codeswitch is primed more by their own speech (i.e., within-speaker priming) than by the speech of others (i.e., between-speaker priming, also referred to comprehension-to-production priming), indicating that priming decreases as a function of the referential distance^2^ between the prime and the target (Fricke and Kootstra, 2016; see also Gries, 2005). Lastly, while spontaneous codeswitching is often deemed characteristic of bilingual discourse, the vast majority of utterances bilinguals produce are unilingual. For example, in the Bangor Miami Corpus, Fricke, and Kootstra reported that of the 42,291 utterances bilinguals produced, the bulk of them (94.2%) were in a single language (see also Beatty-Martínez and Dussias, 2019 for the proportion of unilingual and codeswitched NPs across four bilingual corpora). These factors taken together provide strong evidence that even habitual codeswitchers produce utterances in one language despite high levels of cross-language activation. Thus, bottom-up processes alone, no matter how robust, are not sufficient to account for codeswitching behavior in its entirety. Below, we consider how the speaker’s intentions may exert top-down control over codeswitching practices to achieve communicative goals.

## Codeswitching as a Repair Strategy

The ease of producing speech with little conscious effort and few errors belies the complexity of its underlying cognitive processes. Speech disfluencies (e.g., pauses, false starts, and/or hesitations) are direct evidence of production difficulty (Arnold et al., 2000); the fact that speakers make errors while planning utterances and sometimes correct them evinces the need for monitoring and control in production (Nozari and Novick, 2017).^3^ As a result, speakers may learn implicit strategies to mitigate production difficulty (MacDonald, 2013; Dell and Chang, 2014). Here, we consider the idea that increased cognitive demands in language production may promote codeswitching as a *deus ex machina* of sorts: proficient bilinguals who have extensive codeswitching practice resort to such behavior as a way to mitigate speech planning demands that arise during the normal course of developing a speech plan (e.g., MacDonald, 2013). For bilinguals, speech planning is subject to the parallel activation of the two languages (Kroll et al., 2006), creating many opportunities for cross-language interference, and increasing the potential for within-language interference (Abutalebi and Green, 2007). Bilinguals must, therefore, develop language regulatory strategies to help them manage the relative activation of the two languages when planning goal-oriented speech (Bogulski et al., 2019). Such strategies may include actively suppressing one language to enable fluent speech in the other language when the desire (or requirement) is to use one language alone, but they may also include codeswitching when the desire is to use both languages opportunistically (Green, 2018).

One way to examine this issue is by identifying the types of phonetic and prosodic variation that arise in codeswitched speech. In an analysis of the Bangor Miami Corpus of Spanish-English codeswitching (Deuchar et al., 2014), Fricke et al. (2016) found that lexical items involving a spontaneously-produced codeswitch had reduced speech rate and were more disfluent, relative to matched unilingual control lexical items. To a large extent, one can view these acoustic features as proxies for production difficulty, where slower speech rate and decreased fluency are associated with reduced automaticity (e.g., Segalowitz, 2010). Fricke et al.’s analysis of voice onset time (VOT) further revealed that low-level phonetic modulations often occur in anticipation of a codeswitch: English voiceless stops/ptk/were produced with more Spanish-like VOTs the closer they were to Spanish words, suggesting that these processing costs may more adequately reflect changes in the relative activation of the two languages (see also Balukas and Koops, 2015, for a similar result with codeswitching bilinguals from New Mexico). It is possible that these phonetic changes arise due to the unintended activation of the non-target language, forcing the speaker to switch languages to maintain fluidity in the conversation. Conversely, speakers may have a strong desire to switch languages, and the anticipation of the switch leads to a momentary reorganization of the language system.

To dissociate these two explanations, we turn to a recent study by Johns and Steuck (2018) on the prosodic structure of codeswitched speech in the New Mexico Spanish-English Bilingual (NMSEB) corpus (Torres Cacoullos and Travis, 2018). They observed that codeswitching was more likely to occur toward the end of a prosodic sentence, suggesting that harder-to-produce elements, i.e., those that tend to be produced later in utterances (MacDonald, 2013), will often co-occur with codeswitched speech. Critically, however, they also observed faster speech rates within codeswitched prosodic sentences, relative to unilingual control utterances. This latter finding is important because it suggests that codeswitching is not a source of production costs *per se*. On the contrary, it may help bilingual speakers circumvent difficulties that are inherent to speech planning more generally, hence why it is more likely to occur toward the end of a planned utterance.

It is important to reiterate that, whereas Johns and Steuck (2018) focused on the speech rate within a prosodic sentence, Fricke et al. (2016) focused on the speech rate of words preceding codeswitches. This contrast reveals how codeswitching may come to affect bilingual speech at different levels of planning and raises the question of how to interpret the production costs observed in Fricke et al.’s study. We believe they reflect a momentary reorganization of the prosodic and phonetic systems, and that this reorganization is driven by a deliberate intent to switch languages. From this perspective, codeswitching serves two important functions in production. First, it enables speakers to negotiate lexical competition in a way that minimizes the impact of within-language and cross-language lexical interference. These prosodic and phonetic changes observed within single lexical items may in turn facilitate planning at higher levels, with the goal of maximizing fluency at the discourse level (see Hopp, 2015, 2016, for a similar account on how lexical processing impacts sentence comprehension in bilinguals). Second, the fact that codeswitching leads to systematic variation in speech means that listeners can reliably exploit these cues to facilitate comprehension (Fricke et al., 2016; Guzzardo Tamargo et al., 2016; Valdés Kroff et al., 2017; Beatty-Martínez, 2019; Shen et al., 2020).

## Codeswitching and the Problem of Variable Equivalence

If codeswitching enables bilinguals to successfully navigate linguistic interference in production, what are the strategies that reliably promote a codeswitch? One possibility is that bilinguals rely on cross-linguistic convergence to ensure that a codeswitch is successfully deployed. Research on codeswitching constraints (e.g., the equivalence constraint; Poplack, 1980) and cross-linguistic priming (see section “The contributions of bottom-up factors in codeswitching”) provide some basis for this idea but are insufficient to explain the overall pattern of data available to date. Interestingly, such an account predicts that bilinguals will consistently avoid “conflict sites” (Poplack and Meechan, 1998, p. 132) across the two languages when attempting to switch. But since we have argued that codeswitching is a tool to negotiate speech planning difficulties, we would expect opportunistic use of the languages at sites of variable equivalence, where the languages partially overlap (Torres Cacoullos and Poplack, 2016). One way to tease this apart is by examining the prosodic structure of unilingual and codeswitched speech.

Recent evidence suggests that bilinguals strategically employ prosodic distancing at codeswitch junctures where the two languages sometimes differ due to independent, but inherently variable, processes to execute a codeswitch (Torres Cacoullos and Travis, 2018). Like Johns and Steuck (2018), this area of research examines prosodically-transcribed spontaneous bilingual data where the speech stream is segmented not into boundaries of major syntactic constituents but rather in stretches of speech uttered under a single intonation contour (e.g., intonation units; henceforth, IUs; Du Bois et al., 1993). Prosodic boundaries are perceptually delimited by a set of acoustic features (e.g., a pause, an initial rise in overall pitch level, and final phrase lengthening), and have been presented as evidence that speakers plan their speech in relatively large chunks, corresponding to IUs (Krivokapić, 2012; Bishop and Kim, 2018). Given that it has been argued that speakers plan speech at prosodic boundaries (Krivokapić, 2014), it is likely that linguistic material in the same prosodic unit is planned differently than those occurring in different units.

We illustrate this argument with recent developments in the prosodic positioning of complement clauses. Whereas main clauses typically co-occur in different IUs, main and complement clauses, which share a tighter syntactic relationship, tend to co-occur in the same IU (Du Bois, 1987; Croft, 1995; Steuck, 2016). Steuck and Torres Cacoullos (2019) observed the same pattern in the speech of Spanish-English bilingual speakers when speaking in either of their two languages. Interestingly, main and complement clauses appeared to be prosodically less integrated when bilinguals codeswitched at the clause boundary, a result that could be interpreted as evidence for prosodic distancing (see example 1a below). However, Steuck and Torres Cacoullos also reported that when codeswitching occurred elsewhere (i.e., within the main or complement clause, see example 1b), the rate of prosodic integration of the two clauses was no different than unilingual IUs. Thus, prosodic distancing is not an inherent consequence of codeswitching, but rather serves as a strategy for negotiating cross-linguistic differences between the two languages: the complementizer “that” is present variably in English, while the complementizer “que” is present always in Spanish (Torres Cacoullos and Travis, 2018).


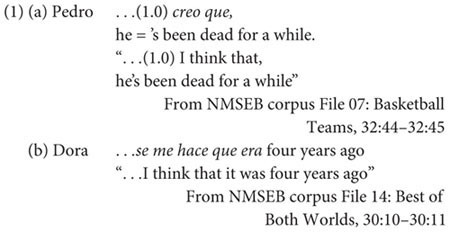


Perhaps most telling is that bilinguals overwhelmingly prefer to codeswitch at prosodic boundaries rather than within IUs despite cross-linguistic differences (Shenk, 2006; Durán-Urrea, 2012; Myslín and Levy, 2015). For example, Steuck and Torres Cacoullos (2019) reported that 60% of codeswitches involving main and complement clauses were at the boundary between the two clauses. Plaistowe (2015) extends this pattern more broadly too: in the NMSEB corpus (Torres Cacoullos and Travis, 2018), speakers switched at IU boundaries 93% of the time. Why might this be? We consider the following possibility: the tendency of codeswitching at IU boundaries may reflect the outcome of a competitive process between active items of both languages and where codeswitching is best understood as an opportunistic response of the most active and most easily retrieved items (Green and Wei, 2014). We infer that the pattern will depend, first and foremost, on how speakers manage the relative activation of their languages, as shaped by their habits of language use and the control demands of their interactional context (Green and Abutalebi, 2013; Green and Wei, 2014; Beatty-Martínez et al., 2019). For example, bilinguals in single-language contexts engage language control competitively (i.e., where language membership is maximized and the activation of one language is suppressed at the expense of the other). In turn, bilinguals in codeswitching contexts engage language control cooperatively (i.e., where language membership is minimized and coactivation is maintained all the way through speech planning so that items from both languages make themselves available for selection).

## Codeswitching as an Opportunistic Strategy

Recently, Green and Abutalebi (2013) and Green and Wei (2014) proposed that bilinguals in a dense-codeswitching context make use of processes related to opportunistic planning (e.g., Hayes-Roth and Hayes-Roth, 1979; Patalano and Seifert, 1997), spontaneously taking advantage of unforeseen opportunities to achieve their communicative goals. Despite growing interest in this idea, there is little empirical research directly examining how bilinguals make use of such a strategy in spontaneous discourse. Below we provide evidence for opportunistic planning by examining the production preferences in the modification of complex NPs of Spanish-English bilinguals living in San Juan, Puerto Rico. Before describing the distributions themselves, we provide a brief overview of the interactional context, participants, and data collection methodology. While Spanish remains the predominant language of Puerto Rico, the use of English is loosely supported in many contexts of everyday life (e.g., in education, media, and other societal domains). Importantly, codeswitching is very common among bilinguals, especially those of the younger generations (Casas, 2016; Pousada, 2017; Beatty-Martínez, 2019; Guzzardo Tamargo et al., 2019). Thus, it follows that bilinguals in this context may be able to use whichever words and structures that are most active to achieve their communicative goals with little-to-no interactional cost (Green and Abutalebi, 2013; Beatty-Martínez et al., 2019). In other words, “their skill lies less in avoiding language conflict than in utilizing the joint activation of both languages and adapting their utterances appropriately” (Green, 2011; p. 2). Codeswitching in this context therefore represents a device for taking advantage of the more efficient of the two languages (Gibson et al., 2019) and through which the cost in time and resources can be minimized.

The data under study here were obtained from the Puerto Rico subset of the Codeswitching Map Task (PR-CMT) corpus (Beatty-Martínez et al., 2018; Beatty-Martínez and Dussias, 2019; Królikowska et al., 2019), a corpus of unscripted, task-oriented dialogs designed to assess codeswitching behaviors in bilingual speakers. The corpus consists of approximately 2.5 h of recordings with 10 Spanish-English bilinguals (6 female). All participants were native Spanish speakers who had acquired Spanish at birth and English either simultaneously or in early childhood. Participants assessed their own proficiency to be equally high in both languages (see Table 1 for a summary of participant characteristics).

**TABLE 1:** Participant self-reported characteristics.

**Measure**	***M***	***SD***	***95% CI***
Age, years	23.3	1.8	22.0–24.6
Spanish proficiency, out of 10	9.6	0.8	9.1–10.1
English proficiency, out of 10	9.6	0.5	9.3–9.9

*Means, standard deviations, and 95% confidence intervals for participants’ self-reported characteristics.*

**FIGURE 1:**
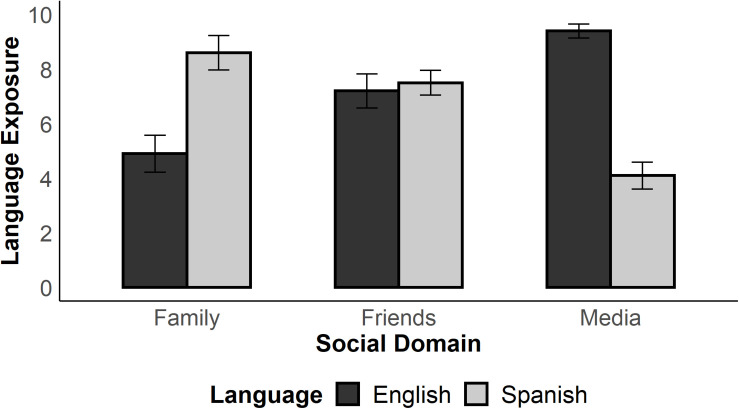
Participants’ self-reported exposure to Spanish and English across different social domains. Ratings were made on a 10-point scale ranging from 0 (no exposure) to 10 (high exposure). Error bars indicate standard error of the mean.

Participants also answered questions about overall language exposure to Spanish and English and their frequency of use in various contexts in daily life. As depicted in Figure 1, participants reported more exposure to Spanish when interacting with family, more exposure to English in the media, but being exposed to both languages equally among friends. Descriptively, these data exemplify how participants’ interactional context supports the use of both languages.

In the map task, director-matcher pairs took turns describing visual scenes (i.e., maps) to one another within a designated time limit. Participants played the role of the director, sitting at a table opposite a confederate matcher who was both a close friend and an in-group member from the same speech community (i.e., San Juan, Puerto Rico). This is important, as previous research has shown that speakers may produce four times as many codeswitches in informal contexts when they are paired with an in-group interlocutor (Poplack, 1983). Furthermore, unlike other guided production tasks where the data distribution is typically controlled and participants are either forced to switch languages or familiarized with object names before the interaction takes place, dialogs were completely unscripted and conversational partners were free to use whichever language they wanted. This sacrifice in experimental control is compensated by the opportunity to offer insights of non-standard language use within the speech community (Sankoff, 1988; Torres Cacoullos and Travis, 2018).

**FIGURE 2:**
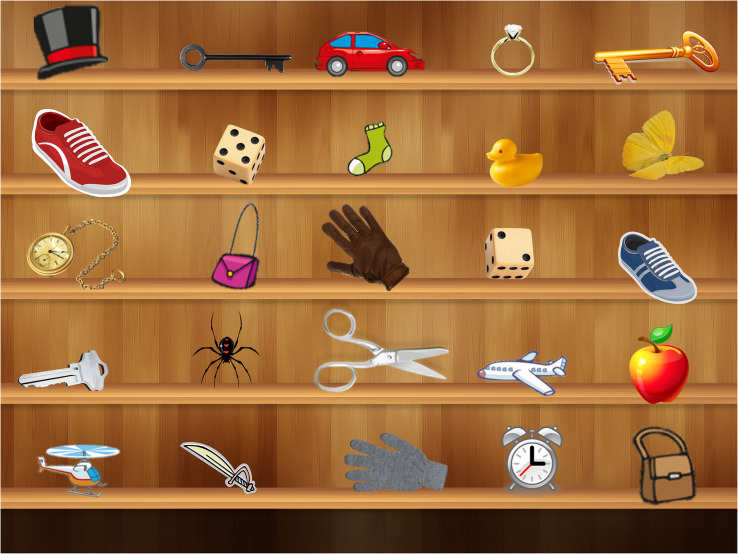
A visual panel from the Codeswitching Map Task.


Director and matcher maps differed only in terms of the way the objects were arranged on a computer screen. Visual scenes contained background objects that were fixed; moveable objects were placed in reference to fixed objects, exerting the need to describe them in terms of their spatial arrangement (see Figure 2 for an example). Visual maps required to replicate the experiment are included as Supplementary Material Files. All objects were presented in color to elicit more detailed descriptions. Additionally, some objects appeared more than once in the same slide, but with different qualities (e.g., a series of faces differing in their facial expressions; see Gullberg et al., 2009; Pivneva et al., 2012; Valdés Kroff and Fernández-Duque, 2017, for similar procedures) as evidenced in excerpt (2) below:

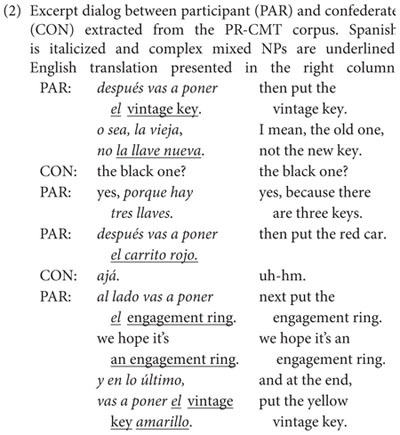


Our quantitative analysis abides by the principle of accountability (Labov, 1972), comparing the rate of codeswitching across different types of constructions by contextualizing them with respect to the contexts where they could have occurred but did not (i.e., by circumscribing the variable context; Labov, 2005). This approach has been widely employed in corpus analyses of codeswitched speech by extracting not only codeswitched tokens across the different types of constructions, but also their unilingual counterparts in Spanish and English (Poplack, 1980, 2017; Torres Cacoullos and Travis, 2018; Steuck and Torres Cacoullos, 2019). Table 3 summarizes the distribution of unilingual and mixed NPs extracted from the corpus. We begin by examining the distribution of simple NPs –composed only of a determiner and a noun– across unilingual and mixed phrases. As shown in Table 2, the vast majority of NPs in the corpus were unilingual (Unilingual, Mixed: χ^2^ = 321.14, *df* = 1, and *p* < 0.001), with roughly half of them produced in Spanish and about a third in English. This finding is congenial to past studies showing that codeswitched utterances constitute a small proportion of corpus data, even in communities where codeswitching is a regular communicative practice (Beatty-Martínez and Dussias, 2017, 2019; Green, 2019). For simple mixed NPs, all but three tokens (“la balloon,” “la guitar,” “the rueda”; English ballon, guitar, and wheel, respectively) were comprised of a Spanish masculine determiner and an English noun, replicating the well-documented asymmetry with respect to grammatical gender and switching direction (Poplack, 1980; Valdés Kroff, 2016; Beatty-Martínez et al., 2018; Casielles-Suárez, 2018; cf. Blokzijl et al., 2017).

**TABLE 2:** Number and proportion of noun phrase utterances across languages in the PR-CMT corpus.

**NP Type**	**Example**	**N**	**Proportion**
Spanish NPs	el carro	437	0.498
English NPs	the car	268	0.305
Mixed NPs	el car	173	0.197
Total		878	1.00

*For mixed NPs, all but three tokens (“la balloon,” “la guitar,” “the rueda” English: ballon, guitar, and wheel, respectively) were comprised of a Spanish masculine determiner and an English noun, replicating the well-documented asymmetry with respect to grammatical gender and switching direction (Poplack, 1980; Valdés Kroff, 2016; Beatty-Martínez et al., 2018; Casielles-Suárez, 2018; cf. Blokzijl et al., 2017).*

**TABLE 3:** Number and proportion of complex Adj + N/N + Adj constructions across languages in the PR-CMT corpus.

**NP Type**	**Example**	**N**	**Proportion**
Spanish	el carro **rojo**	105	0.246
English	the **red** car	187	0.438
Mixed	el **red** car/el car **rojo**	135	0.316
Total		427	1.00

*Modifiers in bold font. Instances in which the determiner and noun were in Spanish and only the modifier was codeswitched (e.g., la carita **sad**, English: “the **sad** face”; *N* = 4) were excluded from all analyses because they stem from different baseline mixed NPs. Thus, all complex mixed NPs had a Spanish determiner and an English noun, but the modifier could be in either English or Spanish and could occur in either prenominal or post-nominal position.*

Next, we examine bilinguals’ structural and language choices in the modification of complex NPs (e.g., the black dog) –a site of variable equivalence between English and Spanish–relative to the mixed Determiner + Noun baseline shown in Table 3. Critically, examining the distributional patterns of complex mixed NPs will allow us to explore whether there are opportunistic behaviors in how codeswitching bilinguals manage to negotiate their two languages.

In English, adjectives typically precede the noun (Adj + N; e.g., the_*Det*_ **yellow**_*Mod*_ house_*N*_). In Spanish, most adjectives are typically placed post-nominally (N + Adj; e.g., la_*Det*_ casa_*N*_ **amarilla**_*Mod*_) although there is a small group of modifiers that occurs prenominally (e.g., quantitative modifiers such as ordinals and cardinals; e.g., la_*Det*_ **primera**_*Mod*_ casa_*N*_, “the first house”). A further cross-linguistic difference is that English makes use of compounding freely and productively (i.e., N + N constructions such as “the **diamond** ring”) whereas compounding in Spanish is much more limited, preferring left-headed noun-prepositional-phrase (N + PP) constructions (e.g., “el anillo **de diamante”**; Liceras et al., 2002; Varela, 2012). Lastly, Spanish differs from English in that Spanish agreement rules require that other grammatical elements (e.g., determiners, adjectives, etc.) match the gender of the noun they modify. Against this background, one possibility is that complex mixed NPs should be generally avoided in contexts that require overt gender marking (e.g., Otheguy and Lapidus, 2003; Balam and Parafita Couto, 2019) or “strictly limited” (Pfaff, 1979, p. 306) due to cross-linguistic differences in word order (for Adj + N and N + Adj constructions) and lexicalization preferences (for N + N and N + PP constructions). If this were the case, we would expect to find a decrease in the proportion of codeswitching in complex NPs relative to the proportion of codeswitching in simple NPs. However, in our data, the opposite is true.^4^

While all-Spanish utterances predominate when bilinguals produce simple (Det + N) NPs (Spanish, English: χ^2^ = 40.034, *df* = 1, and *p* < 0.001; Spanish, Mixed: χ^2^ = 113.39, *df* = 1, and *p* < 0.001), they are not preferred when modifiers (i.e., adjectives) are used (Spanish, English: χ^2^ = 22.469, *df* = 1, and *p* = 1.00; Spanish, Mixed: χ^2^ = 3.504, *df* = 1, and *p* = 0.969), as shown in Table 3. This shift in language choice cannot be due to differences in proficiency or exposure, since Spanish is the native and predominant language of this community of speakers.

One potential explanation, following Myslín and Levy (2015), is that the use of English (participants’ less frequent and therefore more salient language) offers a distinct encoding that signals novel information. Such an account would predict an increase in the use of English within complex mixed NPs across all types of modifiers, regardless of the type of modifier and of the type of construction. An alternative hypothesis, and one that we endorse here, is that speakers will adopt strategies from both languages that are advantageous within a given communicative context. In this case, we would expect speakers to prefer the use of prenominal modification strategies (i.e., Adj+N or N+N constructions), which are overwhelmingly preferred in English but can also appear in Spanish with some types of modifiers (e.g., quantitative modifiers). Such a strategy would help disambiguate between competing sources of information in the map task. For example, when referring to duplicate objects such as the gloves displayed in Figure 2, participants could describe the target glove as having a specific color (e.g., “The **brown/gray** glove” in English or “El guante **marrón/gris**” in Spanish) or as being made of a specific material (e.g., “The **leather/cotton** glove” in English or “El guante **de cuero/algodón**” in Spanish). While it is difficult to determine at which point disambiguation is achieved when using English (i.e., listeners could initially consider other brown/gray items such as the brown purse displayed in the figure), what can be said with more certainty is that for Spanish utterances, disambiguation between the target and non-target gloves cannot be achieved until after the noun is spoken (e.g., el guante **marrón/de cuero**). Therefore, bilinguals’ language and structural choices should favor prenominalization in duplicate contexts to facilitate referent identification (Fukumura, 2018), and thus, optimize task performance.

Indeed, a comparison of the proportion of complex mixed NPs in duplicate against singleton items confirmed that the proportion of codeswitches was greater for duplicate items (Duplicate, Singleton: χ^2^ = 4.588, *df* = 1, and *p* = 0.016). Moreover, as the data in Table 4 show, complex mixed NP constructions were overwhelmingly made up of an English prenominal modifier followed by an English noun (e.g., el red car; Prenominal, Post-nominal: χ^2^ = 50.330, *df* = 1, and *p* < 0.001; English, Spanish: χ^2^ = 47.573, *df* = 1, and *p* < 0.001), suggesting that the use of prenominalization increased across the board. That said, we note that not all complex mixed NPs were opportunistic, as there was a smaller subset of tokens containing Spanish modifiers after the noun (e.g., el car rojo). Importantly, however, the pattern of results reported here is consistent with the distributions reported for Spanish-English bilinguals in Miami (Parafita Couto and Gullberg, 2019)^5^ and Northern Belize (Balam and Parafita Couto, 2019).

**TABLE 4:** Distribution of complex mixed NP modifiers across languages and word order in the PR-CMT corpus.

	**Modifier position**				**Total**	
	**Prenominal**		**Post-nominal**			
**Modifier language**	**N**	**Proportion**	**N**	**Proportion**	**N**	**Proportion**
Spanish	01	0.011	15	1.00	**16**	**0.155**
English	87	0.989	00	0.000	**87**	**0.845**
Total	88	0.854	15	0.146	103	1.00

*As mentioned above, all complex mixed NPs comprised a Spanish determiner and an English noun; the variable context is thus limited to modifier language and modifier position. The one token with a Spanish prenominal modifier (i.e., “la izquierda corner,” Spanish: “la esquina izquierda,” English: “the left corner”) is likely a production error. It is the only instance in this corpus analysis where the modifier does not follow the internal grammar of the language in which it was produced. In all other instances, structural equivalence is maintained across the two languages (Poplack, 1980; Meechan and Poplack, 1995).*

As we mentioned earlier, quantitative modifiers (*N* = 32) occur prenominally in Spanish, and as such, these were examined separately. At this point one could speculate that bilinguals simply prefer to produce complex mixed NPs with English modifiers. However, if prenominalization, rather than the use of English *per se*, is key to bilinguals’ structural and language choices, we should then expect a relative increase in the proportion of Spanish modifiers in complex mixed NPs with quantitative modifiers. And, indeed, this is what we observe in Table 5 (Quantitative, Non-Quantitative: χ^2^ = 46.178, *df* = 1, and *p* < 0.001). Moreover, Spanish modifiers were more prevalent relative to English modifiers in this context (Spanish, English: χ^2^ = 11.281, *df* = 1, and *p* < 0.001), demonstrating that bilinguals will capitalize on the dominant language when it converges with the optimal strategy (i.e., prenominalization).

**TABLE 5:** Distribution of Spanish and English quantitative modifiers in complex mixed NPs in the PR-CMT corpus.

**Modifier language**	**Example**	**N**	**Proportion**
Spanish	el **primer** row	26	0.813
English	el **first** row	06	0.188
Total		32	1.00

*Modifiers in bold font. While grammatical gender is not the focus of this analysis, we note that, for tokens with gender-marked modifiers (20/26 Spanish NPs reported above), all but one (“la cuarta bookshelf”) were masculine marked, including instances where the noun’s Spanish translation equivalent is feminine (e.g., “el primer row,” Spanish: “la primera fila/hilera”; “el último column,” Spanish: “la última columna”). Based on these observations, it appears that the masculine default strategy in gender assignment can be extended to modifiers. We did not scrutinize these data further as they have been analyzed previously as part of a different study (Beatty-Martínez and Dussias, 2019).*

The second pattern of results concerns bilinguals’ structural and language choices in N + N and N + PP constructions. Recall that N + N compounds are highly productive in English but dispreferred in Spanish; the opposite is true for N + PP constructions. Notwithstanding, when bilinguals codeswitch, they are able to opportunistically make use of both Spanish and English strategies. Following the same logic as described above, one possibility is that bilinguals will show a preference for English lexicalization strategies, given that the use of the N + N construction allows the speaker to focus on what is perhaps more important or conceptually salient earlier in the utterance (MacDonald, 2013; Fukumura, 2018). Because Spanish is the dominant language, we can interpret the switch from Spanish into English in mixed N + N constructions as reflecting an opportunistic response, suggesting that the English strategy was most active and most easily retrieved. As Table 6 shows, bilinguals are actively making use of the N + N construction. In all codeswitched tokens, both the head noun and the modifier were produced in English and were preceded by a Spanish masculine determiner. Remarkably, the rate of mixed N + N constructions is nearly identical to that of unilingual English utterances (English, Mixed: χ^2^ = 0.115, *df* = 1, and *p* = 0.367) and is higher than the codeswitching rate reported previously (N + N, Adj + N: χ^2^ = 6.662, *df* = 1, and *p* = 0.005). We speculate that this increase may be related to chunking, the process by which frequently co-occurring sequences of words are grouped together in cognitive representation (Bybee, 2013; Christiansen and Chater, 2016). Because chunking is a gradient phenomenon, Adj + N and N + N constructions (e.g., such as “blue shoe” and “tennis shoe”, respectively) can be conceptualized as falling on a continuum, where instances with stronger collocational associations are more likely to be accessed as a single unit rather than compositionally (Bybee, 2010).

**TABLE 6:** Number and proportion of N + N constructions across languages in the PR-CMT corpus.

**NP Type**	**Example**	**N**	**Proportion**
English	the **diamond** ring	41	0.526
Mixed	el **diamond** ring	37	0.474
Total		78	1.00

*In all codeswitched tokens, both the head noun and the modifier were produced in English and were preceded by a Spanish masculine determiner. Nominal modifiers are presented in bold font. We note that N + N constructions are rare in Spanish occurring only in highly lexicalized expressions (e.g., el perro **policía**; “the police dog”). There were no instances of this type of construction in this data set.*

Consistent with the prediction that bilinguals would capitalize on language structures with prenominal modification, N + N constructions are produced at a much higher rate in the corpus relative to N + PP constructions (N + N, N + PP: χ^2^ = 37.895, *df* = 1, and *p* < 0.001). As shown in Table 7, the majority of N + PP constructions were produced in Spanish (Spanish, Mixed: χ^2^ = 3.062, *df* = 1, and *p* = 0.040). This can be taken as further evidence for how bilinguals are able to accommodate their production choices to optimize task performance. Notwithstanding, we do not take this finding to indicate that bilinguals disregard the use of Spanish-preferred constructions when codeswitching. The few codeswitches that did occur in the corpus are indicative that bilinguals do consider and make use of alternative forms of expression that would be competing in monolingual contexts. We believe that, in this particular communicative context, N + PP constructions serve as a “just-in-time” or *deus ex machina* resource to circumvent potential pitfalls of the speech plan. An important implication is that bilinguals can use (or switch into) one language while the other language stands at the ready as future challenges and opportunities emerge.

**TABLE 7:** Number and proportion of N + PP constructions across languages in the PR-CMT corpus.

**NP Type**	**Example**	**N**	**Proportion**
Spanish NPs	el anillo **de diamante**	12	0.706
English NPs	the pair **of scissors**	01	0.059
Mixed NPs	el corner **de arriba**	04	0.235
Total		17	1.00

*PP in bold font.*

Altogether, these data provide initial empirical support for opportunistic planning during codeswitching. Contrary to the prediction that bilinguals would avoid switching in contexts of variable equivalence due to differences in word order and lexicalization preferences, we observed increased rates of codeswitching despite any potential costs, consistent with Steuck and Torres Cacoullos (2019). This finding also speaks to bilinguals’ intention to codeswitch as a means to achieve their communicative goals. Specifically, we observed that codeswitching bilinguals capitalize on what is most optimal for the current situation (i.e., prenominal modification) by switching languages when circumstances call for such a change. Codeswitching thus may serve as an opportunistic strategy to make use of whatever comes most readily available, all the while conforming to the goals of the speaker.

## Closing Remarks

The studies reviewed here, together with the data we examined, provide critical evidence for the way in which the language system is controlled. In line with contemporary theoretical models of bilingual speech production and language control (Green, 2011, 2018, 2019; Green and Abutalebi, 2013; Green and Wei, 2014), these data support the notion of a cooperative control state, where both languages may openly contribute to production. This stands in contrast with other forms of language use in which language control is engaged competitively and where the “gate” for non-target language items is locked (Green and Wei, 2014, p. 502). Although, research on bilingual language production has shown that bilinguals demonstrate difficulties in language fluency, due perhaps to reduced functional use of the languages (e.g., Gollan et al., 2008), increased cross-language competition (e.g., Sullivan et al., 2018), or limited proficiency (Bialystok et al., 2008), our data suggest that codeswitching might aid language fluency by allowing both languages to remain active and accessible, and therefore providing an alternative means to convey meaning. It remains to be determined what the role of cognitive control is in spontaneous codeswitched speech relative to unilingual speech (Nozari and Novick, 2017). For now, we note that while such flexibility may not be impervious to production costs that arise during normal speech production (e.g., Green, 2019), having the option to either explore or restrict language control states throughout the planning process may potentially alleviate many cognitive demands. In this way, this finding provides support for the more general notion that speakers adopt implicit strategies to mitigate production difficulty (MacDonald, 2013). While the precise mechanisms underlying codeswitching are yet to be fully understood, we hope this will be an active area of research in years to come.

## Data Availability Statement

All datasets generated for this study are included in the article/Supplementary Material.

## Ethics Statement

The studies involving human participants were reviewed and approved by The Pennsylvania State University Institutional Review Board (Approval Number: 34810). The participants provided their written informed consent to participate in this study.

## Author Contributions

All authors equally conceived the theory and hypotheses presented here and wrote the manuscript.

## Conflict of Interest

The authors declare that the research was conducted in the absence of any commercial or financial relationships that could be construed as a potential conflict of interest.

## Footnotes

1 Although cued language-switching studies provide a direct bridge to the more general phenomenon of task switching and non-verbal cognitive control (e.g., Monsell, 2003; Prior and Gollan, 2011; Zhang et al., 2015), whether the same cognitive and neural processes that underlie cued language switching are also deployed for spontaneously-produced codeswitches is an open question. For present purposes, we treat language switching and codeswitching as qualitatively different phenomena, and thus focus exclusively on codeswitching research.

2 Under usage-based approaches, priming effects are typically evaluated in terms of “referential distance” (Givón, 1983; Myhill, 2005, p. 473), where distance is measured in terms of the number of intervening clauses between the target and the previous mention of the referent as well as the presence or absence of intervening human subjects (Torres Cacoullos and Travis, 2018).

3 Recently there have been a number of studies that have examined disfluencies in codeswitched speech while reading aloud (e.g., Gollan and Goldrick, 2016; Gollan et al., 2017; Halberstadt, 2017). However, it is beyond the scope of this article to determine the extent to which the cognitive processes engaged in a reading-aloud paradigm are generalizable to spontaneous speech production (c.f., Guaïtella, 1999).

4 A reviewer raised the possibility that the absence of English-to-Spanish mixed NPs might affect the predictions regarding bilinguals’ production choices. We hypothesize that where codeswitching norms differ, opportunistic strategies may manifest differently. The codeswitching patterns of Nicaraguan bilinguals are an interesting test case as they seem to differ from other Spanish-English bilingual communities, exhibiting a marked preference for English determiners in simple mixed NPs (e.g., “the perro” instead of “el dog”; Blokzijl et al., 2017). Given that prenominal modification is most optimal (in terms of greater discriminatory efficiency), the more opportunistic strategy would be to avoid switching within complex mixed NP structures altogether (preferring unilingual English complex NPs instead). Our hope is that the proposal put forth here will inform and shape future research directions.

5 Note that this study also reports a similar pattern for two other language pairs (Welsh-English and Papiamento-Dutch) with the same conflict regarding the relative order of the adjective and the noun.

## Supplementary Material

PRESENTATION S1 Director slides for the Codeswitching Map Task.

PRESENTATION S2 Matcher slides for the Codeswitching Map Task.
