# Supplementary figures and images for: Codeswitching: A Bilingual Toolkit for Opportunistic Speech Planning
Source: Front Psychol. 2020 Jul 17;11:1699. doi: 10.3389/fpsyg.2020.01699 (PMC7380110; doi:10.3389/fpsyg.2020.01699)

## Slide 1
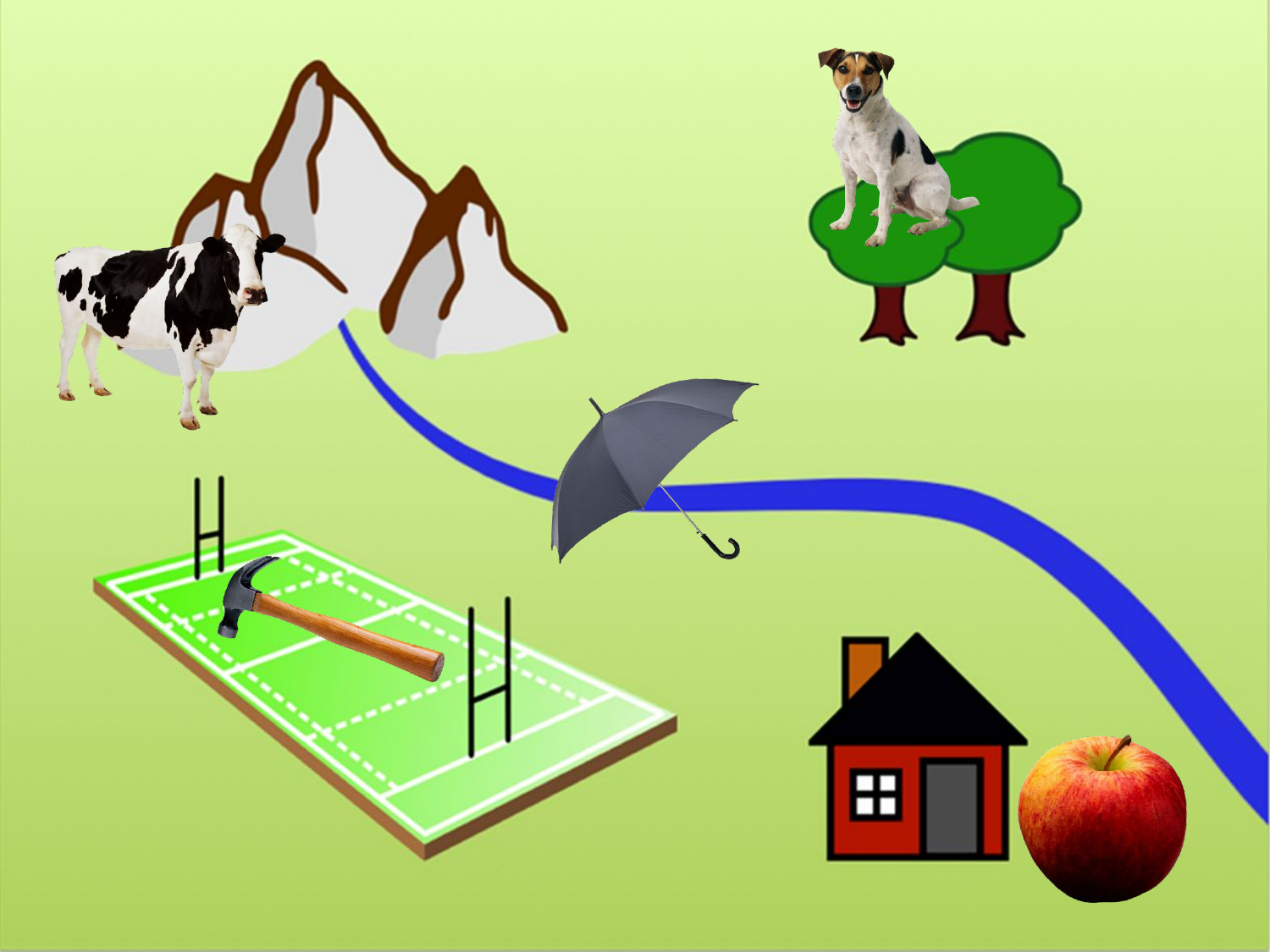

## Slide 2
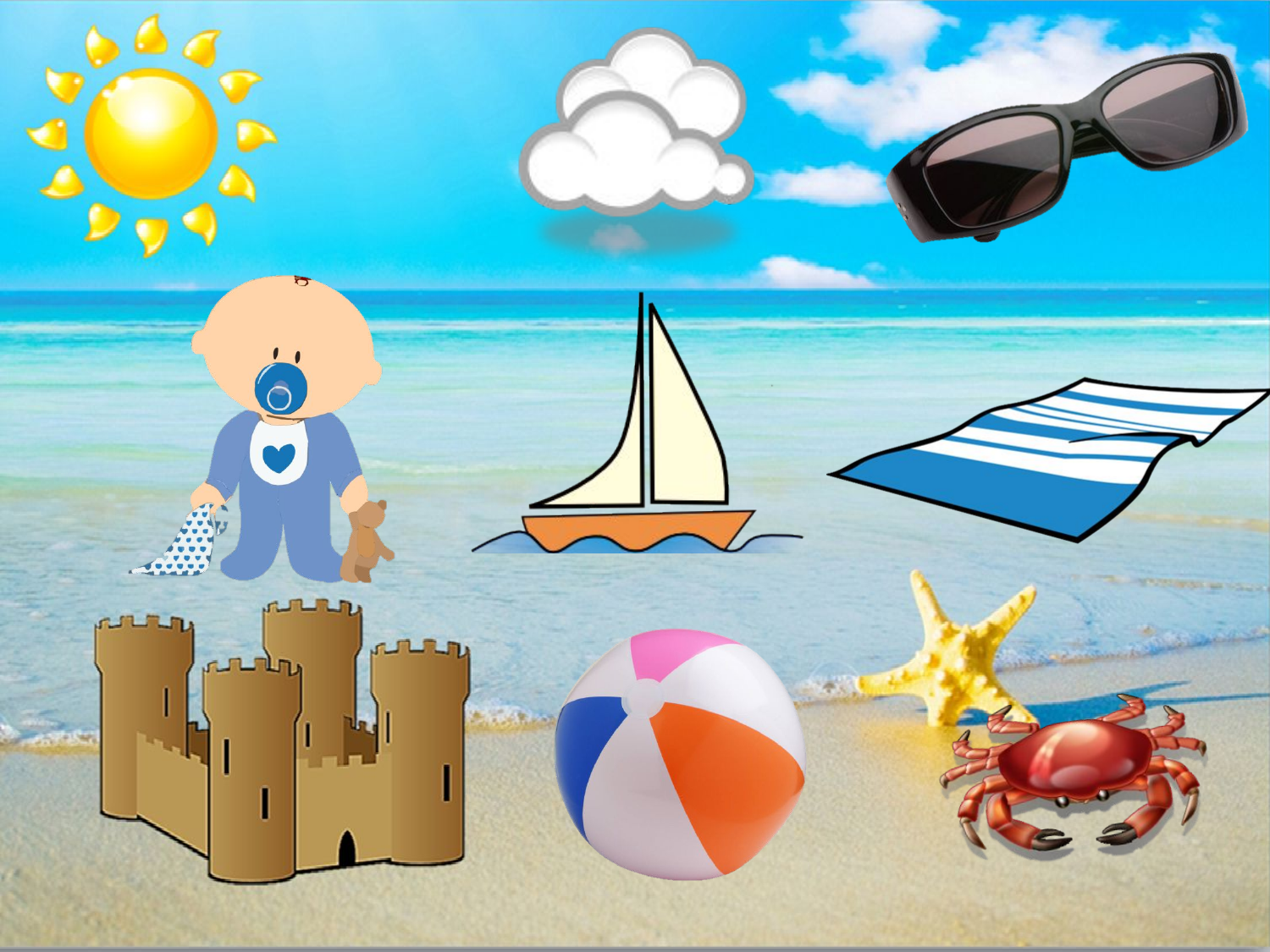

## Slide 3
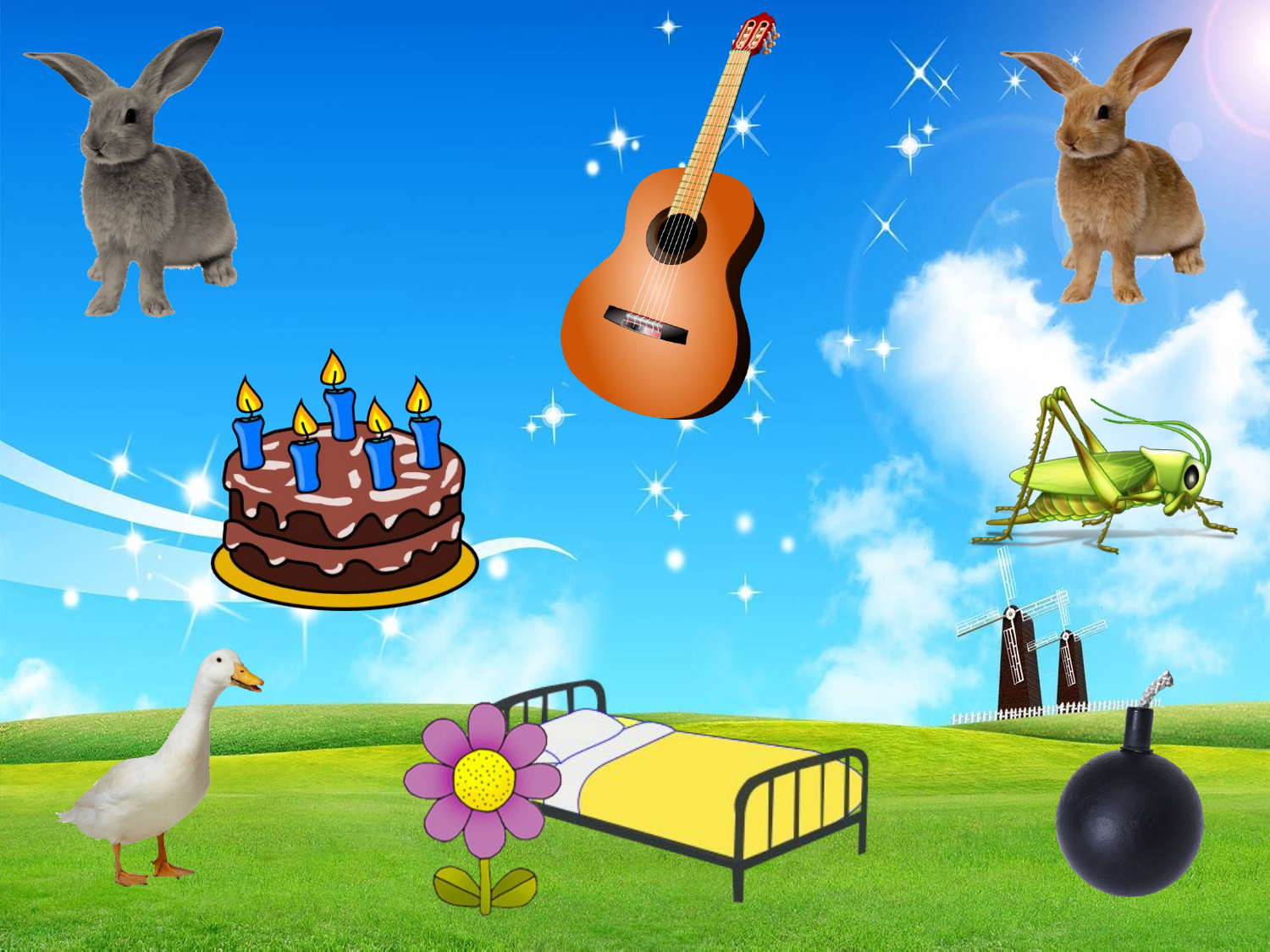

## Slide 4
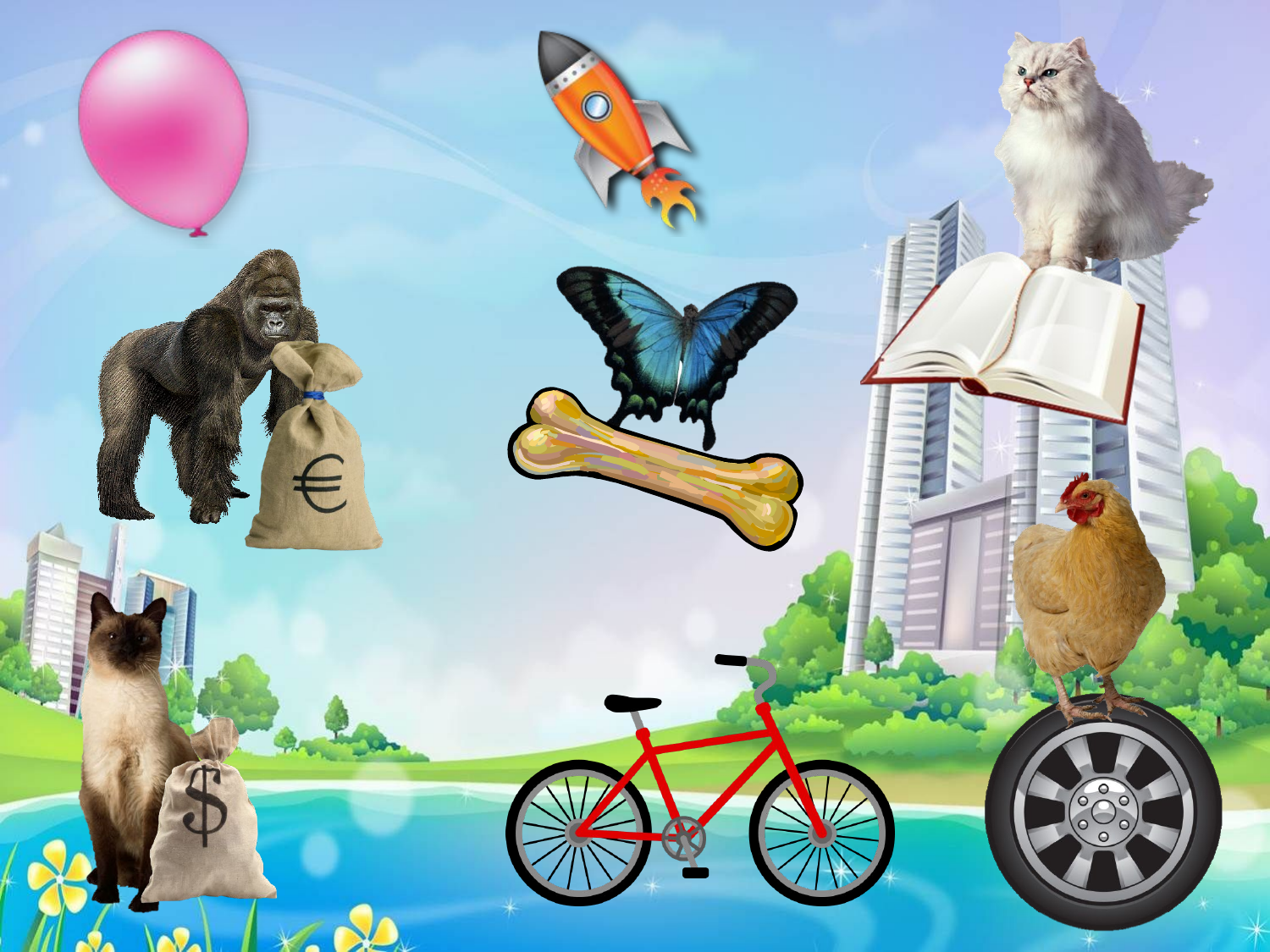

## Slide 5
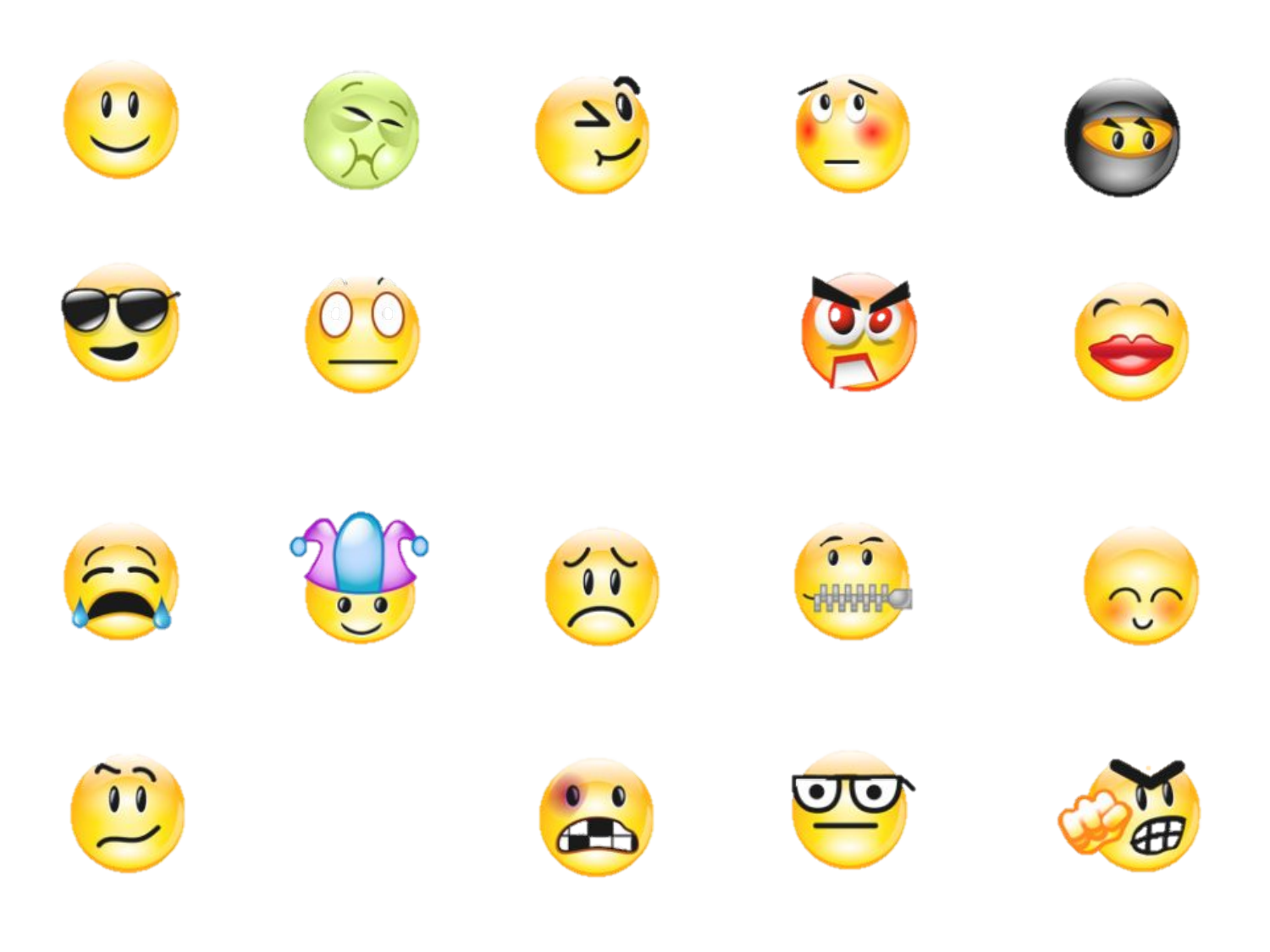

## Slide 6
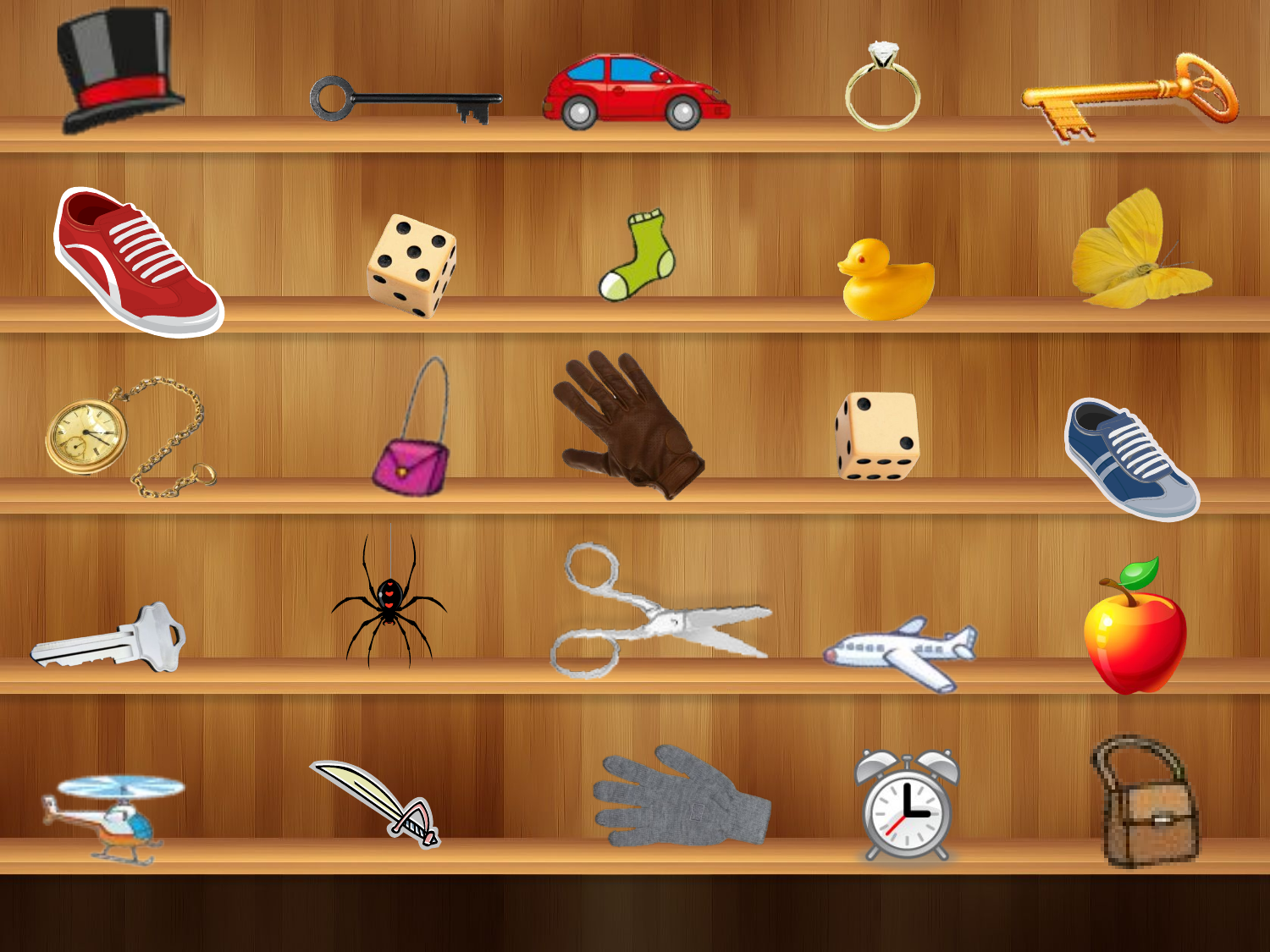

Supplement: PRESENTATION S1 — Director slides for the Codeswitching Map Task. [file Presentation_1.PPTX]

## Slide 1
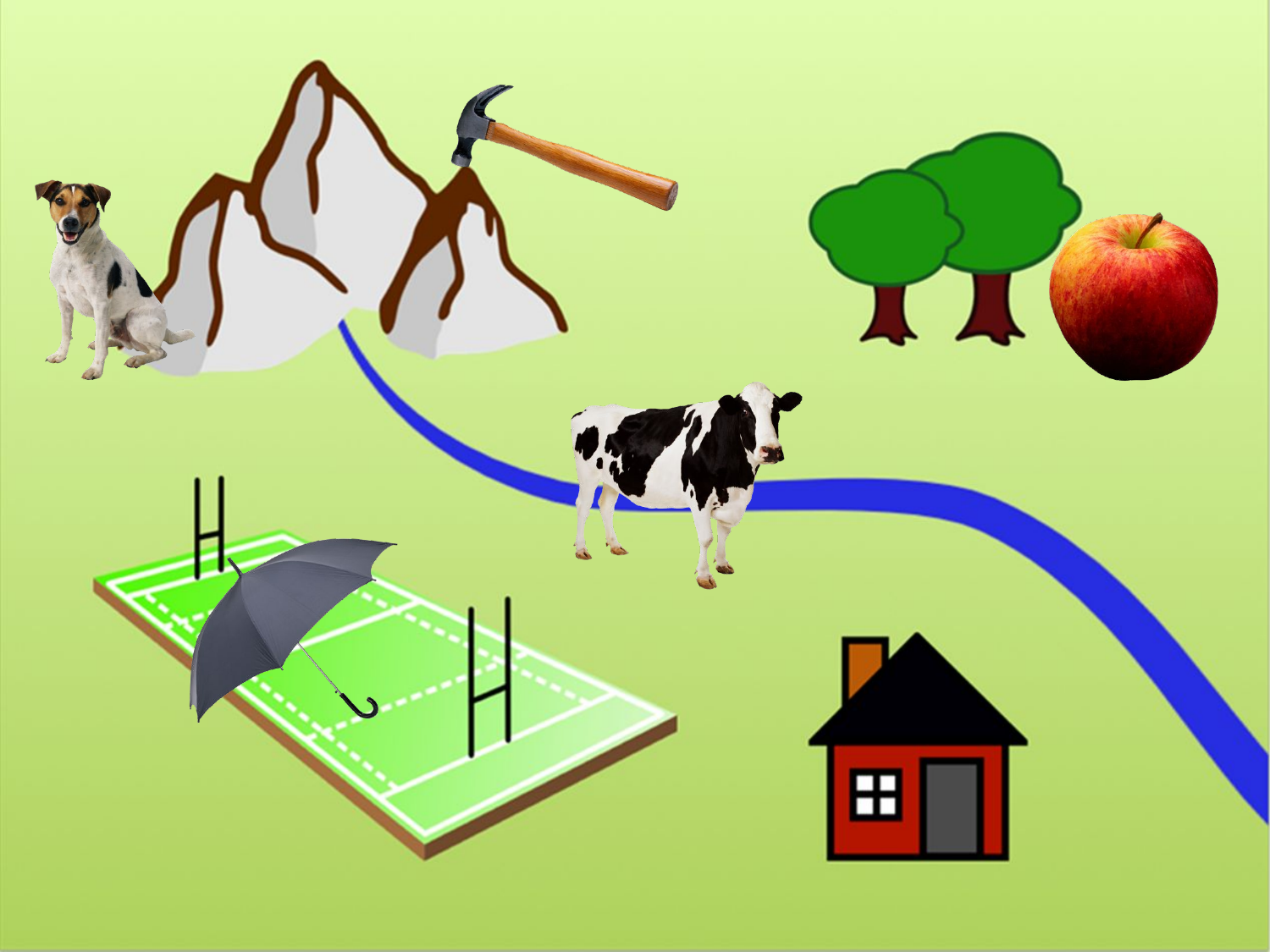

## Slide 2
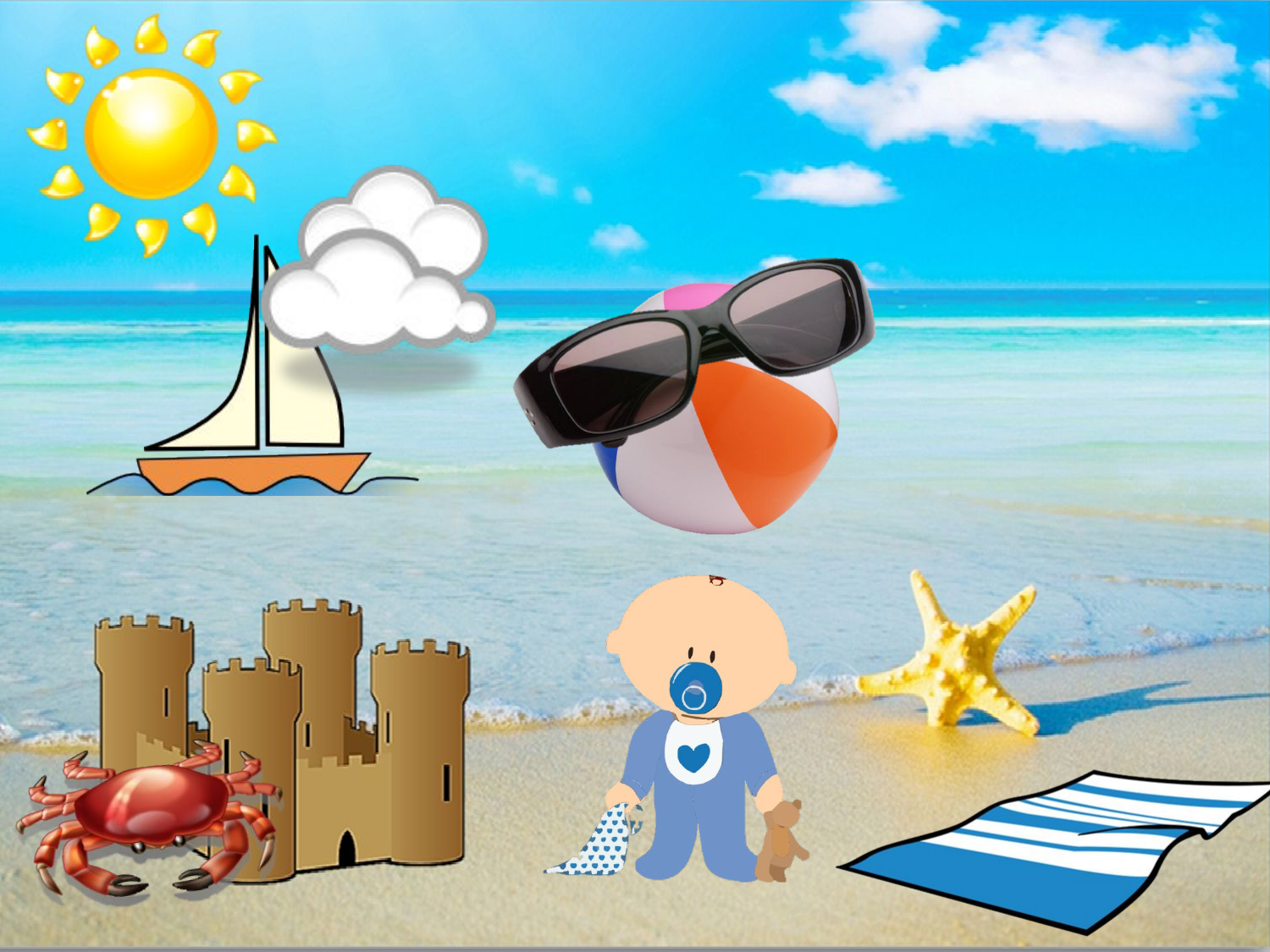

## Slide 3
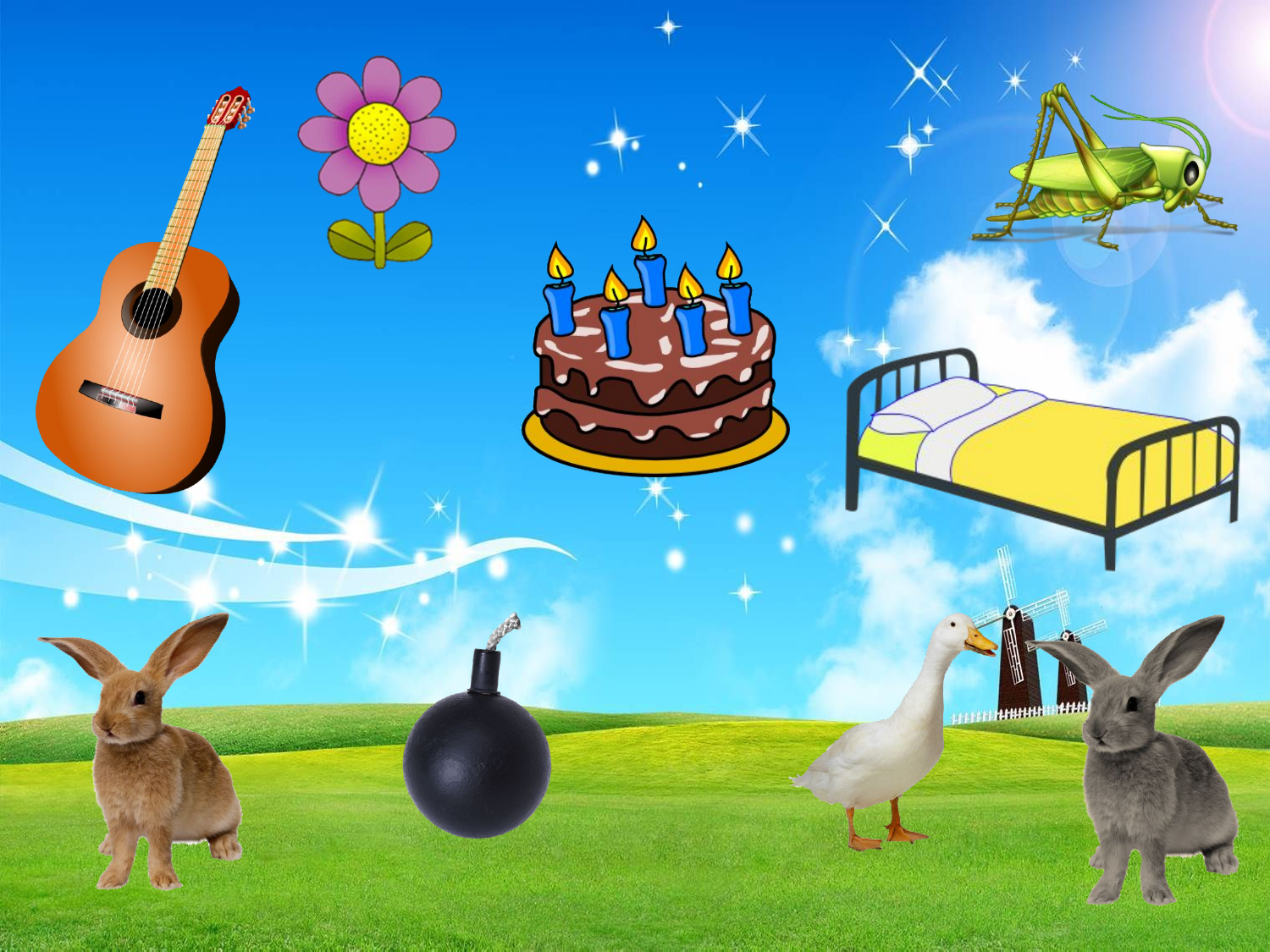

## Slide 4
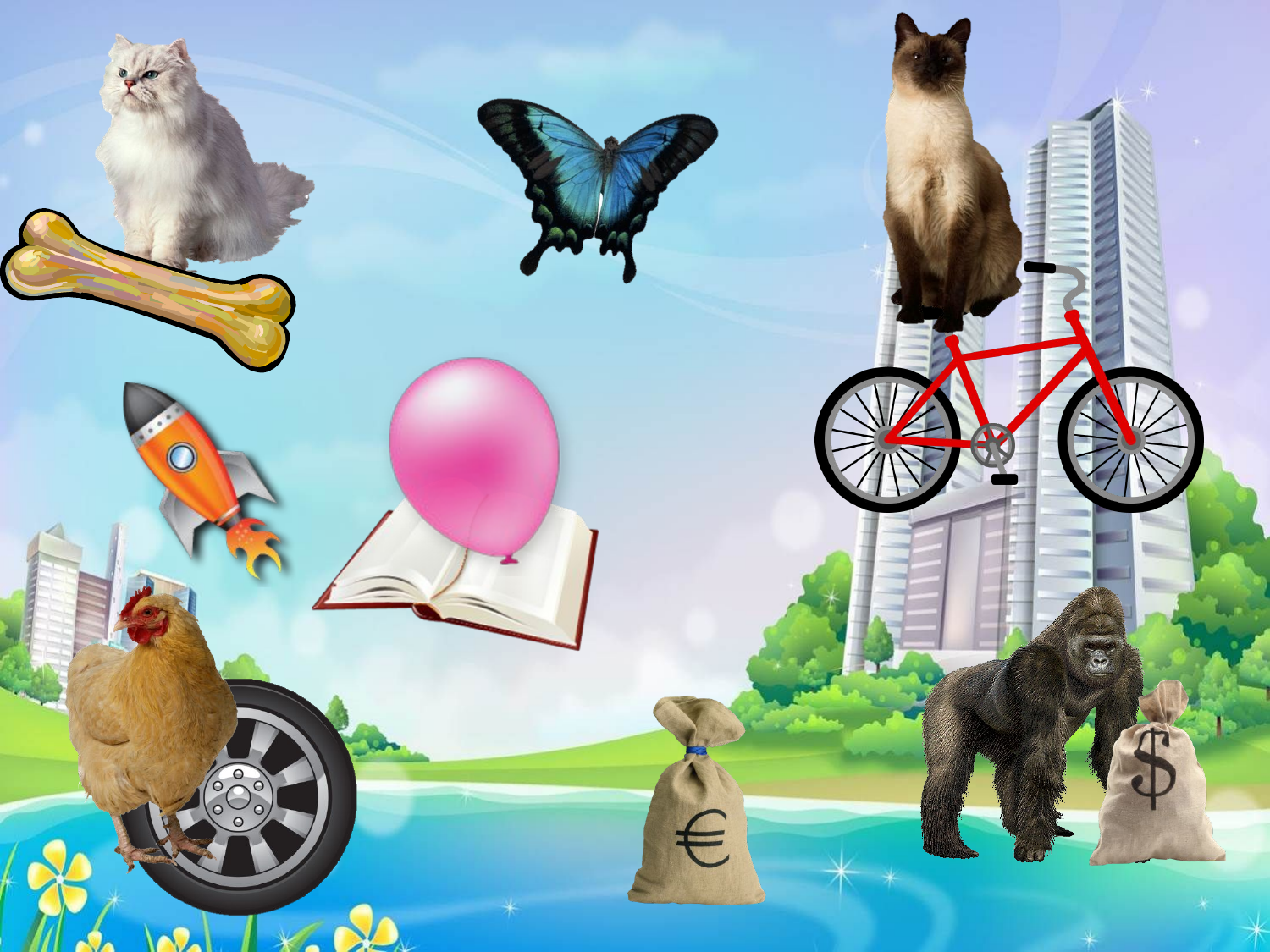

## Slide 5
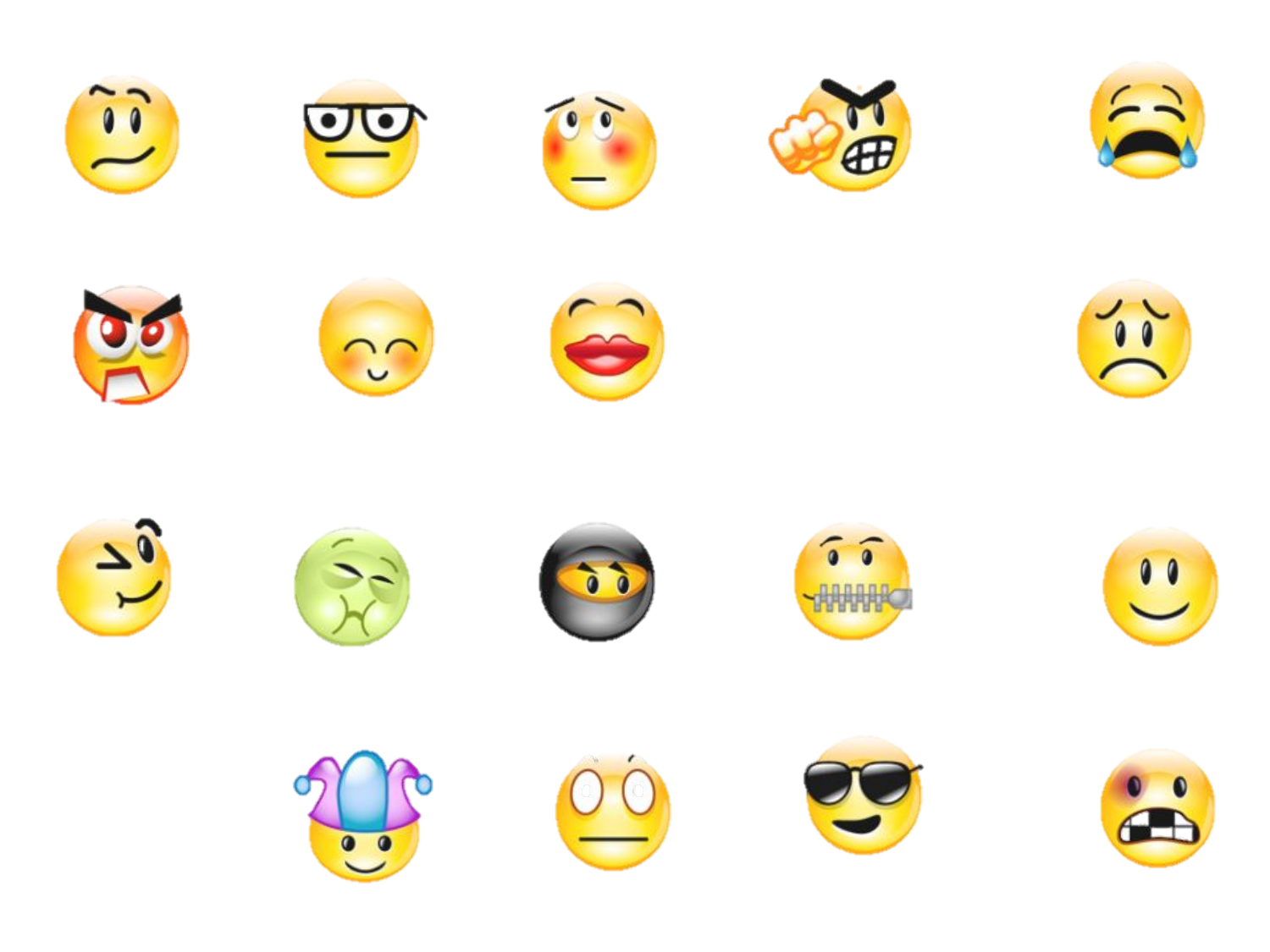

## Slide 6
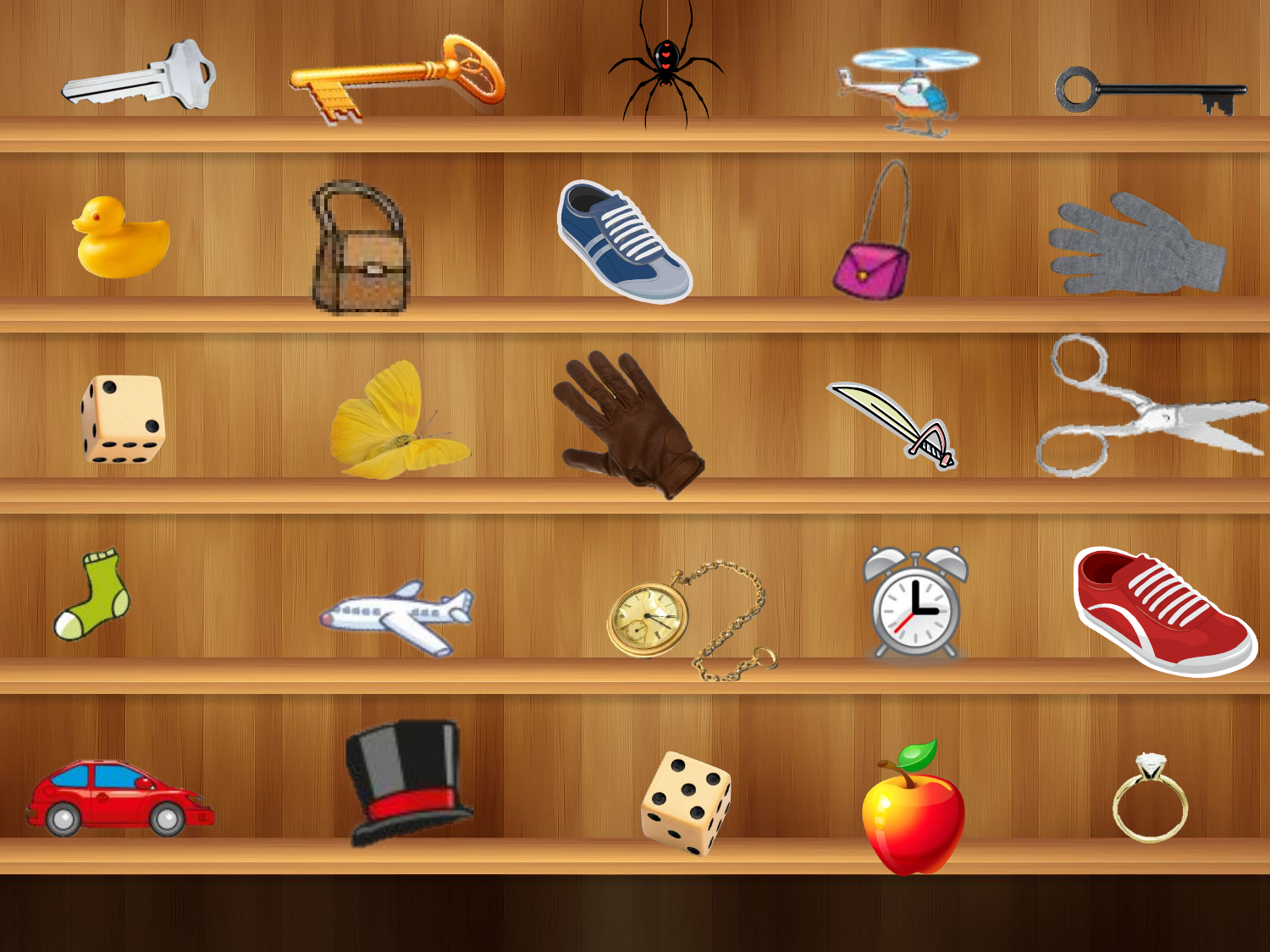

Supplement: PRESENTATION S2 — Matcher slides for the Codeswitching Map Task. [file Presentation_2.pptx]
